# Metal–Organic Framework (MOF) Derivatives as Promising Chemiresistive Gas Sensing Materials: A Review

**DOI:** 10.3390/ijerph20054388

**Published:** 2023-03-01

**Authors:** Huijie Wei, Huiyan Zhang, Bing Song, Kaiping Yuan, Hongbin Xiao, Yunyi Cao, Qi Cao

**Affiliations:** 1 Key Laboratory of Energy Thermal Conversion and Control of Ministry of Education, School of Energy and Environment, Southeast University, Nanjing 210096, China; 2 Frontier Institute of Chip and System, Fudan University, Shanghai 200438, China; 3 Key Laboratory of Optoelectronic Technology and Systems of Ministry of Education, College of Optoelectronic Engineering, Chongqing University, Chongqing 400044, China; 4 Laundry Appliances Business Division of Midea Group, Wuxi 214028, China

**Keywords:** MOF derivatives, ZIFs, MIL frameworks, metal oxides, gas sensors, chemiresistors

## Abstract

The emission of harmful gases has seriously exceeded relative standards with the rapid development of modern industry, which has shown various negative impacts on human health and the natural environment. Recently, metal–organic frameworks (MOFs)-based materials have been widely used as chemiresistive gas sensing materials for the sensitive detection and monitoring of harmful gases such as NO*_x_*, H_2_S, and many volatile organic compounds (VOCs). In particular, the derivatives of MOFs, which are usually semiconducting metal oxides and oxide–carbon composites, hold great potential to prompt the surface reactions with analytes and thus output amplified resistance changing signals of the chemiresistors, due to their high specific surface areas, versatile structural tunability, diversified surface architectures, as well as their superior selectivity. In this review, we introduce the recent progress in applying sophisticated MOFs-derived materials for chemiresistive gas sensors, with specific emphasis placed on the synthesis and structural regulation of the MOF derivatives, and the promoted surface reaction mechanisms between MOF derivatives and gas analytes. Furthermore, the practical application of MOF derivatives for chemiresistive sensing of NO_2_, H_2_S, and typical VOCs (e.g., acetone and ethanol) has been discussed in detail.

## 1. Introduction

With the recent rapid development of cities and industry, volatile organic compounds (VOCs) and other harmful gases are being released at an increasing rate, which upon entry into the air destroys the environmental and ecological balance [1,2]. For example, NO*_x_* causes photochemical smog, SO_2_ causes acid rain and haze, and HCHO causes inflammation and cancer, each of which is harmful and has a noticeable impact on human health [3,4,5,6]. Sensors, which are monitoring devices that convert monitored information into electrical signals or other information, have many advantages, such as simple operation, facile pretreatment, low cost and real-time detection compared with traditional gas analysis instruments such as gas chromatographs and mass spectrometers [7,8,9,10,11,12]. In previous studies, electrochemical sensors, optical fiber sensors, and capacitive sensors have been applied for the detection of harmful gases [13,14,15,16,17]. However, these sensors have a series of disadvantages, such as low sensitivity, complex design, and high prices. Chemiresistive gas sensors, as a kind of promising chemical gas sensor, are ohmic contact resistors with two electrodes displaying resistance changes when sensing materials come into contact with the analyte, and the nature of the chemical reaction and the concentration change of the analyte can be inferred by this resistance change [18,19]. Chemiresistive gas sensors avoid the shortcomings of other types of gas sensors commonly used in previous research due to their several advantages, such as having a fast response speed, high sensitivity, easy-to-design structure, simple operation, and low price; thus, they have been widely used in recent years. It is important to choose gas sensors that are suitable for the recording and monitoring of chemical stimuli and variations in the environment in order to study the changeable trends of harmful gases and in turn set more effective pollution control plans, control the emissions of gas pollutants, and reduce their negative effects. The microstructure and surface state of sensitive materials can influence the performance of gas sensors. Extraordinary structures can increase the number of surface active sites and accelerate the reaction rates [20,21,22,23]. For example, a large contact area caused by hollow or porous structures can increase the degree and rate of chemical reactions. Lee et al. studied the acetone sensing properties of ZnO nanoparticles with different oxygen vacancies [24]. The study showed that the existence of oxygen vacancies brought about an excellent response because controlling the surface defects represented a way to change the sensing properties. O_2_ in the air adsorbs on the surface of ZnO nanoparticles and combines with the electrons above to form O_2_^−^, wherein the oxygen vacancy can be used as an electron donor [25,26,27,28,29,30]. When the sensor is exposed to acetone, O_2_^−^ reacts with acetone, and the adsorbed electrons are then released back into the conduction band (CB), resulting in the reduction of the depletion layer thickness and resistance. Thus, it is important to choose a suitable sensitive material.

Metal–organic frameworks (MOFs), a burgeoning class of metal-coordinated cationic polymer nanomaterials consisting of metal cation aggregates which are linked by organic ligand molecules or metal cations, have large specific surface areas, tunable and porous structures, and high structural stability. Furthermore, MOFs also show excellent adsorption capacity and low environmental toxicity, making them promising candidates for environmental applications such as adsorption and catalysis [31,32]. However, MOFs are generally electrical insulators with poor conductivity. This greatly hinders their usage for chemiresistive gas sensing materials due to the foreseeable intrinsic low response values. In this case, pristine MOFs have been combined with other conductive materials to form heterostructures, either doped with heteroatoms, or simply calcined at high temperature to generate metal oxide-carbon composites (i.e., MOF derivatives) to improve their sensing performance [33,34]. For example, a uniform three-dimensional MXene/MOF composite, Ti_3_C_2_T*_X_*/ZIF-67/CoV_2_O_6_, has been obtained by a co-precipitation reaction, which integrated the conductivity of MXene and the redox activity of the MOF [35]. Generally, MOFs are suitable precursor materials which can be used as an ideal template to synthesize porous metal nano-oxides with an ideal structure. In this regard, their derivatives, such as metal oxides, carbon materials, and their composites, are often used as gas sensing materials due to their high chemical stability, rich structural diversity, large specific surface areas with highly porous structures, considerable temperature resistance, low environmental and physiological toxicity, and many other advantages [19,36,37,38,39]. Furthermore, the doping of heteroatoms into MOFs is also a method for improving sensing performance. Doping heteroatoms can reduce the particle size of the sensing material by inducing the formation of oxygen vacancies, increasing the contact area and enhancing the sensing performance [40,41,42,43]. In addition, the ion or compound activation centers formed by doping heteroatoms can be used as catalysts in chemical reactions between detected gases and sensing materials, accelerating reactions and improving the selectivity and response speeds of sensing materials at a low temperature. Thus, doping heteroatoms into metal oxide-based sensing materials is beneficial for improving their gas-sensing properties [33,34,35,36,37,38,39,40,41,42,43,44,45,46,47]. For example, Zhang et al. synthesized Cu–In_2_O_3_ hollow nanofibers by doping Cu atoms into In_2_O_3_ and used them as H_2_S-sensing materials. Their study showed that the doping of Cu atoms and the formation of their hollow structure increased the number of active sites, and the small amount of CuO formed by Cu oxidation could combine with In_2_O_3_ to synthesize the *p*–*n* heterojunction, greatly improving selectivity and responsiveness to H_2_S [48]. In addition, the doped heteroatoms could also accelerate the adsorption and desorption of oxygen to electrons, thus improving response performance [49]. In this paper, we summarize the methods of enhancing the sensing performance of chemiresistive gas sensors for harmful gases, such as by changing the morphology and structure of MOFs, doping heteroatoms, and other design methods.

## 2. Sensing Principles of Chemiresistive Gas Sensors

The chemiresistive gas sensor, an important part of the gas analysis system, is an instrument which can determine the concentration and composition of detected gas and convert the obtained chemical information into electrical information [50,51]. Chemiresistive gas sensors are often used to detect inorganic small molecular gases and VOCs. Chemiresistive gas sensors react differently when exposed to different inorganic small molecule gases. If exposed to a gas with strong oxidizing or strong reducing properties, the substances in the sensor and the analyte exchange electrons and holes or form a heterojunction to affect the resistance. If exposed to acid and alkaline gas, the substance in the sensor affects the resistance through the occurring chemical adsorption and chemical reaction with the analyte [52]. VOC detection mostly uses polymer as the gas-sensing material of the chemiresistive gas sensor. If exposed to a non-conductive polymer, the sensitive film begins to expand, the conductive path becomes longer, electron transfer becomes more difficult, and the resistance value increases after the polymer absorbs VOCs. However, the resistance value is changed via electron-hole exchange, forming a heterojunction if exposed to conductive polymers [53,54,55,56,57,58]. In previous studies, the performance of chemiresistive sensors has usually been evaluated by the following parameters: (1) Response (*R*): *R* = (*R*_air_ − *R*_gas_)/*R*_gas_, where *R*_gas_ and *R*_air_ are the resistance with and without the presence of gaseous analyte; (2) Sensitivity (*S*): *S* = δ*R*/δ*C*_t_, where *C*t is the concentration of the measured gas; (3) Repeatability: Repeatability is measured by comparing the response of the same device to the same concentration of measured gas for multiple cycles; (4) Stability: Stability refers to testing the gas-sensing performance of the same device to the measured gas after being placed at different times, comparing the change in gas sensitivity with time; (5) Selectivity: Selectivity of the gas sensor is evaluated by comparing the response gap of the same device to different gases with the same concentration.

## 3. Chemiresistive Gas Sensors Using MOF Derivatives

MOF derivatives-based chemiresistive sensors have attracted wide attention. They could simply be synthesized by thermochemical methods such as pyrolysis, using MOF as the template compromising the different functional units including metal ions and carbon species [59].

### 3.1. NO_2_ Sensors

**Figure 1:**
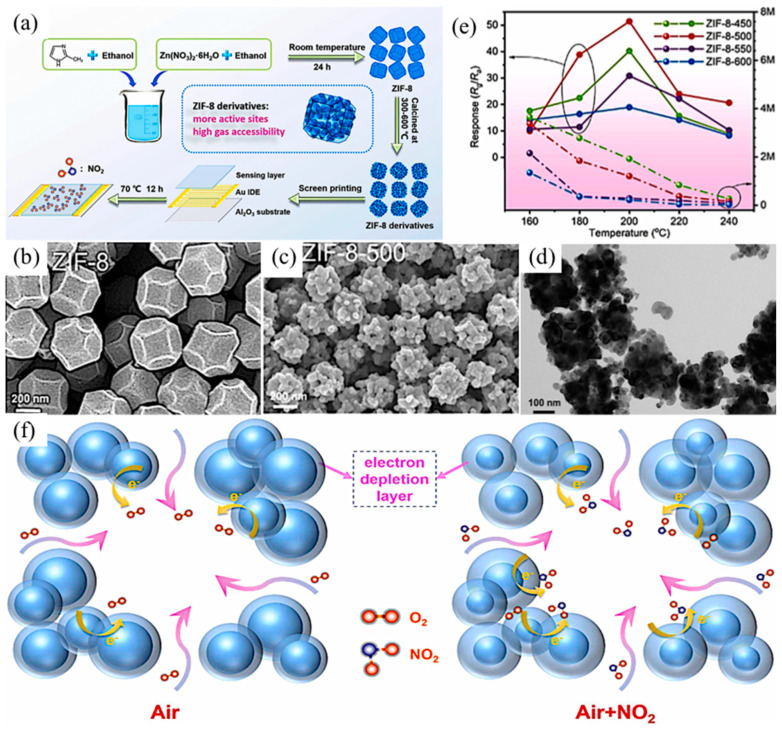
(**a**) Schematics of the synthesis and fabrication process of a ZIF-8 derivatives-based sensor; SEM images of (**b**) ZIF-8 and (**c**) ZIF-8 derivatives after 500 °C pyrolysis; (**d**) TEM images of porous ZIF-8-500; (**e**) Response value to 1 ppm NO_2_ at different operating temperatures of ZIF-8-derivatives after different pyrolysis processes; (**f**) Mechanism for the NO_2_ sensing process of ZIF-8-500 [65].

NO_2_, a kind of hazardous vehicle emission product and combustion product of fossil fuels with an acidic nature, can cause many environmental problems, such as acid rain, photochemical smog, haze, and water eutrophication [60,61,62]. Thus, it is very important to develop gas sensors with high sensing performance for NO_2_ detection. Studies to date have shown that nanostructured metal oxides have higher sensing performance for NO_2_ [63,64]. Ren et al. used a Zn-based zeolitic imidazolate framework (ZIF-8) as a template to synthesize porous ZnO nanocubes for the detection of NO_2_ (Figure 1a) [65]. The experiment indicated that as the temperature increased, the organic bonds of the compounds were gradually pyrolyzed, the organic ligands were removed, and the metal nodes were oxidized to metal oxides leaving voids, as demonstrated by scanning electron microscopy (SEM) images of ZIF-8 and ZIF-8 derivatives at different pyrolysis temperatures (Figure 1b,c). By comparing the response of ZIF-8 derivatives synthesized at different pyrolysis temperatures to 1 ppm NO_2_, it was noted that ZIF-8-500 synthesized at the pyrolysis temperature of 500 °C had the highest response (Figure 1e). Previous studies have confirmed that the charge transfer between absorbed gas and the gas sensor may affect the sensing performance of metal oxide gas sensors. When the gas sensor is exposed to NO_2_, NO_2_ adsorbs on the material and takes electrons from the CB of ZnO, causing a resistance increase and producing an electron depletion layer (Figure 1f). Porous ZnO nanocubes inherit the high specific surface area of ZIF-8, and its unique porous hollow polyhedral structure creates numerous gas channels, making it easier for NO_2_ to adsorb on its surface and take away electrons. Compared with normal ZnO, porous ZnO nanocubes present high sensitivity and a lower NO_2_ detection limit. In addition, the prepared gas sensor exhibits good selectivity for NO_2_ compared with other gases (CO, C_2_H_5_OH, H_2_, H_2_S, NO, NH_3_). This is attributed to the excellent microstructure and surface states of the nanomaterials.

**Figure 2:**
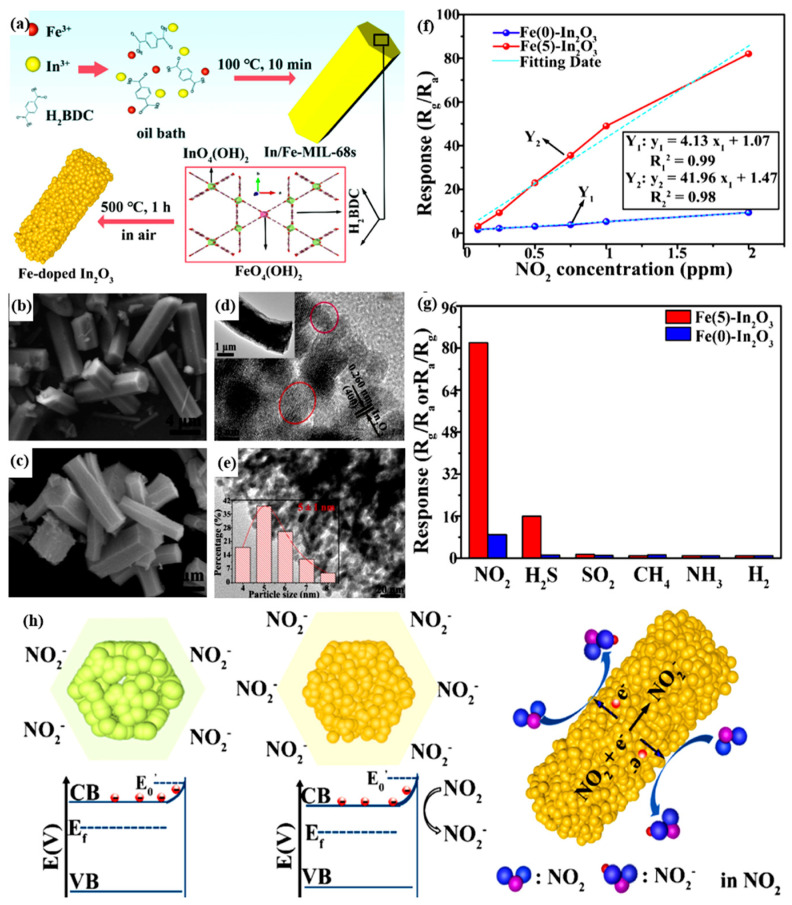
(**a**) Synthetic process of the mesoporous Fe-doped In_2_O_3_ nanorods derived from In/Fe-MIL-68s; SEM images of (**b**) In/Fe-MIL-68s precursor with 5 mol.% Fe(III) content and (**c**) as-resulted Fe(5)–In_2_O_3_ nanorods; TEM images of as-resulted (**d**) Fe(0)–In_2_O_3_ and (**e**) Fe(5)–In_2_O_3_ porous nanorods; (**f**) Response of Fe(0)–In_2_O_3_ and Fe(5)–In_2_O_3_ porous nanorods toward different concentrations of NO_2_ at 80 °C; (**g**) Selectivity of the Fe(5)–In_2_O_3_ sensor toward various tested gases with the concentration of 2 ppm at 80 °C; (**h**) Schematics for the sensing mechanisms of pristine In_2_O_3_ and Fe-doped In_2_O_3_ nanorods [44].

Materials Institute Lavoisier (MIL)-based materials synthesized from terephthalic acid and metal-centered octahedron (MO_4_(OH)_2_, M = In, Ga, Fe), which are organic ligands, have a three-dimensional network structure with ultra-high porosity [66]. Du et al. took MIL (M = In) as a precursor doped with a small amount of Fe ions to synthesize Fe–In_2_O_3_ nanorods through thermochemical methods and a pyrolysis process, investigating its NO_2_ sensing performance (Figure 2a) [44]. By observing the SEM images of In/Fe-MIL-68s with different levels of Fe(III) content, all the samples showed a hexagonal rod-shaped architecture, the Fe-doped In_2_O_3_ nanorods had pores on the surface, and the structural size was slightly reduced, which was due to the shrinkage and decomposition of the MOF structure caused by the pyrolysis process. In comparing the high-resolution transmission electron microscopy (HRTEM) images of Fe(0)–In_2_O_3_, it was found that doping Fe(III) forms lattice defects because Fe(III) replaces the In^3+^ ions in the crystal structure (Figure 2d,e). According to Figure 2f,g, Fe(5)–In_2_O_3_ nanorods showed excellent responsiveness and selectivity to NO_2_ compared with similar products because the nanorod exhibits superior NO_2_ gas-sensing performance at low temperatures due to the high diffusivity, multiple active sites, and wide depletion layer brought by its unique structure. When the sensor is exposed to air, O_2_ adsorbs on the surface of the nanorods and extracts the electrons in the sensing material, forming O^−^ or O^2−^, which leads to the formation of a potential barrier by bending the energy band and increasing resistance, in turn reflecting the sensing performance of the material for NO_2_. When the sensor is exposed to NO_2_ gas, it contacts and combines with the electrons of the sensing material to form NO_2_^−^, resulting in further band bending, higher potential barrier formation, and the further increase in resistance (Figure 2h). In summary, in order to better adsorb and sense the NO_2_ molecules, the MOF precursors with high specific surface areas should be selected, and the resulting metal oxide composites are usually doped to improve conductivity, and thereby to improve the electron exchange and sensitivity toward NO_2_.

With the rapid development of electronic products, wearability and high selectivity have attracted significant attention. In a study by Bag et al., a NO_2_ sensor based on reduced graphene oxide (rGO)–ZnFe_2_O_4_ was developed by uniformly anchor smearing MOF-derived mesoporous ZnFe_2_O_4_ microparticles on the rGO sensor layer [67,68]. Because of the synergistic reaction between the mesoporous ZnFe_2_O_4_ particles and the rGO sensing layer, the gas sensor showed improved mechanical stretchability and signal stability compared with rGO-only devices, exhibited superior response and sensitivity to NO_2_, and had good repeatability and selectivity even under high humidity conditions, which is expected to be applied in future wearable electronics.

### 3.2. Acetone Sensors

**Figure 3:**
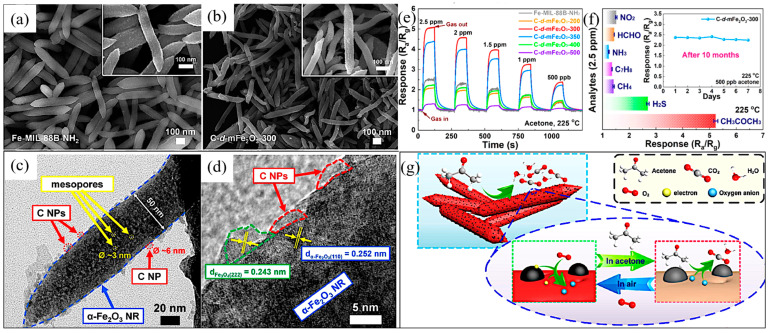
Low- and high-magnification (inset) SEM images of (**a**) Fe-MOF precursor (Fe-MIL-88B-NH_2_ NRs) and (**b**) MOF-derived products obtained under 300 °C; (**c**) TEM and (**d**) HRTEM images of as-obtained carbon nanoparticles decorated mesoporous α-Fe_2_O_3_ NRs (C-d-mFe_2_O_3_-300 NRs); (**e**) Dynamic response curves of gas sensors based on Fe-MIL-88B-NH_2_ and C-d-mFe_2_O_3_-*x* (*x* = 200, 300, 350, 400, and 500) NRs facing different concentrations (0.5–2.5 ppm) of acetone at 225 °C; (**f**) Responses of C-d-mFe_2_O_3_-300 sensor to various gases including NO_2_, H_2_S, NH_3_, C_7_H_8_, CH_4_, HCHO, and CH_3_COCH_3_ with the same concentration of 2.5 ppm; (**g**) Schematic illustration of the acetone sensing process by C-d-mFe_2_O_3_-300 NRs [72].

Acetone, a harmful VOC, is widely used in the chemical industry and chemical experiments. Trace amounts of acetone gas can cause great harm to the environment and to human health, including headache, coma, and even death. Moreover, acetone is one of the important indicators used for diabetes detection [69,70,71]. Thus, it is very important to develop efficient and sensitive acetone gas sensors. Zhu et al. used Fe-MIL-88B-NH_2_ as a precursor and MEMS as a substrate to synthesize Fe_2_O_3_/C mesoporous nanorods (NR) via simple hydrothermal and pyrolysis reactions, and investigated its acetone-sensing properties [72,73]. The experiment indicated that when the calcination temperature was lower than 500 °C, as the temperature increased, α-Fe_2_O_3_ was gradually formed in the compounds, the crystallinity was increased, and the organic molecules were gradually carbonized and decomposed, as shown in the SEM images of the Fe-MOF precursors and their derivatives at different calcination temperatures (Figure 3a,b). Furthermore, the specific surface area of the mesoporous NRs increased significantly after calcination at 300 °C, which was beneficial to their acetone gas-sensing properties. In addition, a hollow structure was formed in the compound, which might have been caused by the lattice rearrangement and the complete decomposition of the organic ligands when the calcination temperature reached 400 °C, as displayed in the TEM images (Figure 3c,d). When the synthesized material was exposed to acetone, the electrons on the Fe_2_O_3_ surface were captured by the oxygen adsorbed on it, resulting in an increase in the depletion layer and the potential barrier. The oxygen species adsorbed on its surface reacted with acetone, changing the resistance (Figure 3g). The study showed that the synthesized carbon nanoparticle-modified mesoporous α-Fe_2_O_3_ NRs exhibited excellent thermal stability, accurate selectivity, and a fast response to acetone (Figure 3e,f), which may have been due to its large specific surface area and excellent electrical conductivity.

**Figure 4:**
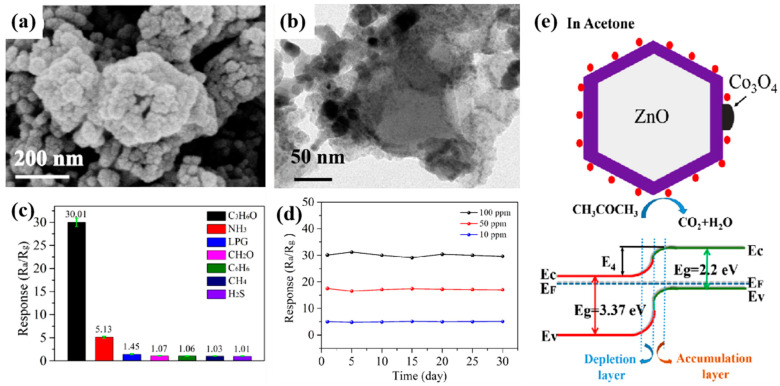
SEM (**a**) and TEM (**b**) images of the ZIF-8/ZIF-67-derived ZnO/Co_3_O_4_ nanopolyhedrons; (**c**) Selectivity of the ZnO/Co_3_O_4_ nanocomposite sensor; (**d**) Long-term stability over 30 days toward 10, 50, and 100 ppm acetone of the ZnO/Co_3_O_4_ nanocomposite sensor; (**e**) Schematics of the acetone sensing mechanism of the ZnO–Co_3_O_4_ heterojunction [74].

Zhang et al. synthesized a ZnO/Co_3_O_4_ nano-heterostructure using ZIF-8/ZIF-67 as a precursor via a facile co-precipitation method [74]. The experiment results demonstrated that ZnO nanopolyhedrons are composed of many small pores. ZnO/Co_3_O_4_ nanopolyhedrons exhibit a similar hollow polyhedron structure with ZnO nanopolyhedrons revealed by the SEM and TEM images (Figure 4a,b). The response diagrams of the ZnO and ZnO/Co_3_O_4_ thin film sensors to acetone were further observed (Figure 4c,d) in the experiment, which showed that the ZnO/Co_3_O_4_ thin film sensor exhibited good selectivity, reproducibility, repeatability, and stability to acetone. When ZnO is exposed to air, oxygen molecules adsorb on the surface of ZnO nanopolyhedrons and obtain electrons to form O^2−^ and O^−^ ions, forming a further depletion layer. When ZnO was exposed to acetone, the acetone reacted with the adsorbed oxygen ions, thereby reducing the height and width of the depletion layer and further reducing the resistance of the sensor (Figure 4e). The sensing mechanism of the ZnO/Co_3_O_4_ acetone sensor is similar to that of ZnO, while the sensing performance is greatly improved with regard to response, repeatability, and selectivity. This may be facilitated by the superior catalytic performance of Co_3_O_4_, the *p*–*n* heterojunction formed between ZnO and Co_3_O_4_, and the unique hollow structure. In general, sophisticated metal oxide-based heterostructures with enhanced porosity and lattice rearrangement, or with a favorable *p*–*n* heterojunction, could be obtained from designed MOFs as the template, which could consequently achieve a rapid response to acetone as a result, being attributable to the improved carrier dynamics [75,76,77,78].

### 3.3. Ethanol Sensors

**Figure 5:**
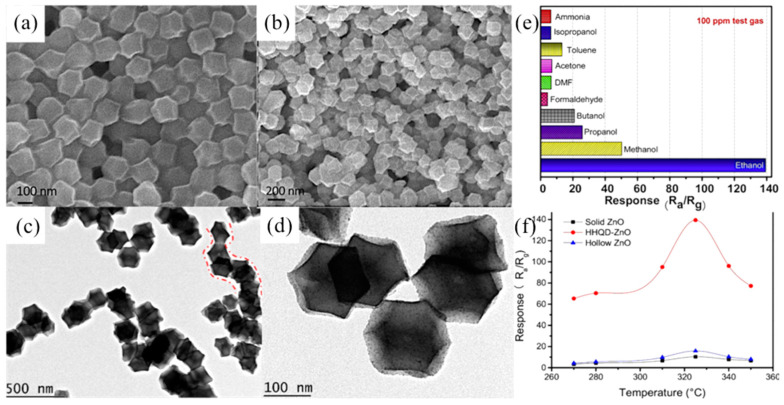
SEM and TEM images of (**a**,**c**) the 170-ZIF-8 precursor and (**b**,**d**) the as-derived HHQD-ZnO nanocage; (**e**) Response of HHQD-ZnO nanocages-based gas sensor toward 100 ppm different target gases (ammonia, formaldehyde, dimethylformamide, acetone, toluene, isopropanol, methanol, propanol, butanol and ethanol, respectively); (**f**) Responses of the sensors based on HHQD-ZnO, hollow ZnO and solid ZnO to 100 ppm ethanol gas at different operating temperatures [80].

Zhang et al. attempted to synthesize solid, hollow, and hierarchical hollow nanocages with quantum dots (HHQD) of ZnO for ethanol gas sensing [79]. The HHQD-ZnO nanocages were synthesized from the ZIF-8 product with a size of 170 mm (170-ZIF-8) as a precursor, and the SEM images of the 170-ZIF-8 nanocrystals (Figure 5a,b) indicated that the synthesized 170-ZIF-8 precursors had a uniform cage-like morphology with good connectivity between particles. The further study of the TEM images revealed (Figure 5c,d) that the formed HHQD-ZnO nanocages inherited the cage-like morphology of the precursor, presenting a large-area hollow structure and a large specific surface area. According to the response diagram of the HHQD-ZnO nanocages to ethanol gas (Figure 5e), the response value of the HHQD-ZnO nanocage sensor to ethanol was much higher than that of the solid ZnO nanocage sensor, and the sensor showed high selectivity to ethanol gas (Figure 5f), which was due to the unique hollow interpenetrating nanostructure and large specific surface area. When the HHQD-ZnO nanocage sensor was exposed to ethanol, its hollow nanocage structure could adsorb oxygen and carry away free electrons from the CB, thereby increasing the potential barrier and resulting in resistance change.

### 3.4. H_2_S Sensors

**Figure 6:**
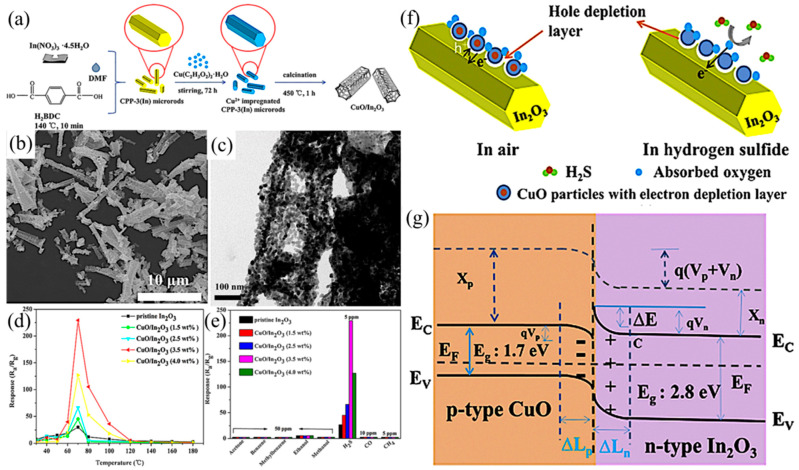
(**a**) Schematic illustration of the formation of CuO/In_2_O_3_; (**b**) SEM image of as-prepared samples of CuO/In_2_O_3_ (CuO concentration = 3.5 wt%) and corresponding (**c**) TEM image; (**d**) Response of each sensor to 5 ppm H_2_S at different operating temperatures; (**e**) Selectivity of each sensor to 5 ppm H_2_S gas and other gases (5, 10, or 50 ppm) at 70 °C; (**f**) Schematics of carrier transportation and gas-sensing mechanism of the CuO/In_2_O_3_ heterostructure; (**g**) Energy level diagram of CuO/In_2_O_3_ heterostructure [88].

H_2_S is a flammable and toxic gas which can be produced in the production processes of food processing plants, paper mills, oil refineries, and other factories, and can also be produced by gas combustion and after the decomposition of human and animal excreta [80,81,82,83,84]. H_2_S can affect the human nervous system and visual system, causing Alzheimer’s disease, loss of consciousness, and other problems [85]. Thus, it is urgent that H_2_S detection and monitoring sensing systems are improved in order to reasonably control H_2_S emissions [86,87]. Li et al. first synthesized CPP-3 (In) microrods and then used Cu^2+^-impregnated CPP-3 (In) microrods impregnated with Cu^2+^ as MOF precursors to synthesize bamboo-like CuO/In_2_O_3_ heterostructures via heating, cooling, centrifugation, drying, and calcination (Figure 6a) [88]. SEM (Figure 6b) and TEM images (Figure 6c) of CuO/In_2_O_3_ showed that the synthesized CuO/In_2_O_3_ inherited the rod-like shape of the CPP-3(In) precursor, but the average size was slightly reduced, showing a bamboo-like hollow structure. In addition, the images also showed the existence of *p*–*n* heterojunctions formed between CuO and In_2_O_3_ nanoparticles. By comparing the response values of CuO/In_2_O_3_ to different gases (Figure 6d,e), CuO/In_2_O_3_ showed excellent selectivity and responsiveness to H_2_S at low temperatures. Intrinsically, CuO is a *p*-type compound and In_2_O_3_ is an *n*-type compound. When CuO came into contact with In_2_O_3_, holes in the CuO and electrons in the In_2_O_3_ flowed in opposite directions, forming an internal electric field, an energy band bending in the depletion layer, and subsequently forming a *p*–*n* heterojunction, enhancing gas-sensing performance (Figure 6f,g). Finally, the unique mesoporous bamboo-like hollow structure and the facilitated electron transfer resulted from the *p*–*n* heterojunction, and together enabled the CuO/In_2_O_3_ sensor superior selectivity, reproducibility, and sensitivity to H_2_S. Furthermore, Karuppasamy et al. synthesized Ni_4_Mo/MoO_2_@C composite nanospheres via a co-precipitation and high-temperature calcination process using MOF as a precursor [89]. The gas response of the Ni_4_Mo/MoO_2_@C composite nanospheres to H_2_S was 3.5 times and 2.6 times higher than those of Ni-MOF and Mo-MOF, respectively, which was attributed to the synergistic effect of the Ni_4_Mo/MoO_2_@C composite nanospheres and the high surface area derived from the unique morphology. Overall, the *p*–*n* heterojunction with well-defined energy level bending can be introduced into the depletion layer with the reverse flow of holes and electrons, which could afford a reliable detection of H_2_S at a lower power consumption [90,91,92].

### 3.5. Other Gas Sensors

Zhang et al. synthesized NiFe_2_O_4_ nano-octahedrons via the direct pyrolysis of NiFe-bimetallic MOFs to explore its gas sensing performance to toluene [93]. The study showed that the sensor exhibited a low recovery time, strong stability, high repeatability, and low detection, which was due to the catalytic properties and high specific surface area resulting from its porous structure. Qin et al. synthesized Co_3_O_4_ dodecahedrons by calcining a Co-MOF template. The sensing material exhibited good selectivity and high responsiveness to CO because of the large number of Co^3+^ active sites and surface adsorption of oxygen [94].

## 4. Conclusions and Future Perspectives

The chemiresistors, a type of chemical gas sensor, can detect the concentration changes of target gases through changes in resistance signals. Studies have shown that the performance of chemiresistors could be promoted by selecting suitable gas-sensing materials to achieve high responsiveness and selectivity to the specific target gas. MOF derivatives synthesized with MOFs as precursors hold great potential in gas sensing due to their unique structures, high selectivity, sensitivity, and versatility. In this review, we summarized the applications of MOF derivatives for sensing NO_2_, acetone, ethanol, H_2_S, and several other toxic gases, with their detailed sensing performance and mechanisms being described and discussed.

However, although the novel MOF derivatives-based chemiresistors suggest many new opportunities in in situ detection and monitoring of the harmful gases in industries and in daily life, there are still several challenging points which need to be dealt with:

(1). The MOF derivatives should successfully maintain or inherit the original high porosity and redox activity of pristine MOFs during the high-temperature pyrolysis process so that they can achieve excellent sensitivity and response as a chemiresistive gas sensor;
(2). The sophisticated morphologies and precisely tailored physicochemical properties of the MOF derivatives need to be constructed and established by thermochemical or other methods, avoiding from the unfavorable Ostwald ripening process, in order to increase the active surface areas affording adsorption and catalysis reaction with gaseous molecules, and also the surface electron affinity to enhance their resistance changing signals;
(3). Efficient charge transfer needs to be realized by the construction of a *p*–*n* junction and other heterojunction interfaces so that rapid response and recovery times are available for the chemiresistive gas sensors;
(4). The reproducibility and cost control for the preparation of MOF derivatives and the as-resulted chemiresistive sensing devices are still far from satisfactory;
(5). The realization of self-powered, minimized and potable gas sensing devices based on MOF derivatives is another indispensable future research direction, especially with the rapid development of 5G wireless networks currently taking place;
(6). Finally, the rapid and efficient data transmission and establishment of the gas sensors-based IoT system, which are of great significance to safer and cleaner production by avoiding the leakage of toxic, harmful, flammable and explosive gases like methane leakage during the exploitation of oil and natural gas, also require the further utilization and optimization of the MOF derivatives-based chemiresistive gas sensors.

## Data Availability

Not applicable.
